# Plant as an Alternative Source of Antifungals against *Aspergillus* Infections: A Review

**DOI:** 10.3390/plants11223009

**Published:** 2022-11-08

**Authors:** Lee Fang Tan, Vi Lien Yap, Mogana Rajagopal, Christophe Wiart, Malarvili Selvaraja, Mun Yee Leong, Puay Luan Tan

**Affiliations:** 1 Faculty of Pharmaceutical Sciences, UCSI University, UCSI Heights 1, Jalan Puncak Menara Gading, Taman Connaught, Cheras, Kuala Lumpur 56000, Malaysia; 2 Institute for Tropical Biology & Conservation, Universiti Malaysia Sabah, Kota Kinabalu 88400, Malaysia

**Keywords:** *Aspergillus*, aspergillosis, opportunistic infections, antifungal activity, plants, plant extracts, bioactives, essential oils

## Abstract

*Aspergillus* species consists of a group of opportunistic fungi that is virulent when the immunity of the host is compromised. Among the various species, *Aspergillus fumigatus* is the most prevalent species. However, the prevalence of fungal infections caused by non-fumigatus *Aspergillus* has been increasing. Polyenes, echinocandins and azoles are the three main classes of antifungal agents being used for the treatment of aspergillosis. Nevertheless, the incidence of resistance towards these three classes has been rising over the years among several *Aspergillus* spp. The side effects associated with these conventional antifungal agents have also limited their usage. This urges the need for the discovery of a safe and effective antifungal agent, which presents a major challenge in medicine today. Plants present a rich source of bioactive molecules which have been proven effective against a wide range of infections and conditions. Therefore, this present review intends to examine the current literature available regarding the efficacy and mechanism of action of plant extracts and their compounds against *Aspergillus* spp. In addition, novel drug delivery systems of plant extracts against *Aspergillus* spp. were also included in this review.

## 1. Introduction

Fungal diseases were estimated to cause about 1.6 million deaths per year while more than 1 billion people were diagnosed with severe fungal diseases [1]. Yet, they are still an under-discussed topic by public health authorities, with a lack of funding supporting the surveillance systems for the monitoring of fungal disease incidence and antifungal drug resistance [2]. Fungi can infect crop plants resulting in a great impact and loss of food crops production thus leading to increased insecurity issues in global food production [3]. Fungal diseases are also of veterinary and medicinal importance and can affect significantly the health and morbidity of animals and humans [4]. The main fungi causing diseases in humans are *Aspergillus*, *Candida*, *Cryptococcus*, *Pneumocystis jirovecii* and endemic dimorphic fungi. In general, humans are relatively resistant to invasive fungal diseases. However, in immunocompromised patients and HIV-infected patients, the susceptibility toward opportunistic fungal infections is increased. *Candida* spp. are the most common isolates from immunocompromised patients, but *Aspergillus* or *Cryptococcus* may also be isolated from these patients [5]. This category of patients includes those who undergo chemotherapy or immunosuppression as well as organ transplantation and even patients with chronic diseases such as diabetes mellitus, AIDS and cystic fibrosis [6,7,8].

*Aspergillus* spp. is one of the most common causes of life-threatening fungal infection, especially in immunocompromised patients. Most infections involving *Aspergillus* spp. are caused by *Aspergillus fumigatus*; however, infections caused by non-fumigatus *Aspergillus* species, such as *Aspergillus flavus*, *Aspergillus niger* and *Aspergillus terreus,* have also been identified [9,10]. Due to the difference in the antifungal susceptibility profile between the different species, it is therefore important to know the distribution and epidemiology of the different species causing infections in different areas in the world to aid in the selection of the most appropriate therapeutic agent [10,11].

At present, three types of antifungal agents are used by clinicians for the treatment of fungal infections, namely polyenes, echinocandins and azoles. Resistance has been an issue due to the limited antifungal agents available while some fungi can be resistant to all three types of antifungal agents [12,13]. In addition, the gold standard antifungal agent used to treat systemic infections, amphotericin B, is also well known to be associated with nephrotoxicity. Its side effect and toxicity may be so severe that discontinuation of therapy is required regardless of having a life-threatening fungal infection [14]. The increase in the incidence of fungal infections, development of resistance and limitations of the currently available antifungal agents increase the need for the discovery of new antifungal agents, ideally with a different mechanism of action.

Natural products play an important role in medical therapy. From January 1981 to September 2019, 1,881 new drugs were approved of which natural products constitute 4.6% while drugs derived from natural products constitute 18.9% [15]. Many natural products, their extracts and secondary metabolites have been found to be effective as anti-inflammatory agents, anti-diabetic, anti-cancer, etc. [16,17]. The pharmacological activities of plants are largely due to their secondary metabolites, inter alia, terpenoids, alkaloids, flavonoids and other phenolic compounds, including tannins, which have been found to exert in-vitro antimicrobial activity [18]. Growing bodies of evidence have demonstrated the potential of higher plants in exerting antimicrobial activity. For example, betel leaf against *Candida* spp. [19] and turmeric against *Schistosoma mansoni* [20,21]. The use of natural products may also provide a better resolution for patients who are afraid of the side effects of conventional synthetic antimicrobial agents. Hence, we intend to discuss the existing literature available regarding the efficacy and mechanism of different plants and plant products in exerting their antifungal activity towards *Aspergillus* spp.

## 2. *Aspergillus* Infections

*Aspergillus* infections are among the most common fungal diseases worldwide. There are several types of *Aspergillus* infections that have been diagnosed, which include but are not limited to allergic bronchopulmonary aspergillosis, chronic pulmonary aspergillosis, aspergillus spondylitis, invasive aspergillosis and cerebral aspergillosis. *Aspergillus* infections can infect both healthy and immunocompromised individuals, although infections are more common in the immunocompromised, and are often associated with a bad prognosis [22]. Invasive aspergillosis is the most severe form of aspergillosis, in which the infection spreads from the lungs to other distant organs. The increased spread of the new severe acute respiratory syndrome coronavirus 2 (SARS-CoV-2) causes dysregulation of the immune system, facilitates infection by *Aspergillus* spp., leading to COVID-19-associated pulmonary aspergillosis (CAPA) [23]. Patients with underlying haematological disorders, undergoing immunosuppressive therapy and those having chronic illnesses such as diabetes mellitus, chronic kidney disease (CKD) and chronic obstructive pulmonary disease (COPD) are at a higher risk of developing *Aspergillus* infections [6,7,8]. Recent global estimates have also reported 3 million cases of chronic pulmonary aspergillosis, while more than 300,000 cases were identified for invasive aspergillosis [22]. The total direct medical costs caused by *Aspergillus* infections were estimated to be $1,254,833,662 in the United States in 2017 [24]. The total hospitalization costs inflicted by *Aspergillus* infections (total cost of $1.2 billion) were also the second highest among all the other fungal diseases [24].

*Aspergillus* infection is caused by *Aspergillus* spp., which consists of more than 250 species, with fewer than 40 species being associated with infections in humans. *A. fumigatus* is the main species causing infection in humans and is the main contributor to death among infected patients. However, non-fumigatus *Aspergillus* species such as *A. flavus*, *A. niger* and *A. terreus* have also been isolated from patients with invasive aspergillosis [25]. The reason why *A. fumigatus* remains the major pathogenic agent as compared to other species is unclear; however, it could be associated with its resilience in withstanding a wide range of environmental conditions in comparison to other species. In this context, *A. fumigatus* is able to tolerate a wide range of temperature and pH in which it can be isolated from decaying vegetation and soil with a temperature range of 12 °C to 65 °C and pH of 2.1 to 8.8 [26]. In addition, the presence of genes encoding glycosylhydrolases and a group of extracellular proteinases allow *A. fumigatus* to survive the degradation of polysaccharides from plant cell walls and nitrogen sources acquired from the degradation of proteinaceous substrates [26,27]. These reasons could have enhanced the ability of *A. fumigatus* to survive in the environment; however, its association with the enhanced pathogenic potential as compared to the other species will require further exploration and evidence.

## 3. Current Treatment for Aspergillosis

Despite the discovery of several antifungal agents for the treatment of mycoses, their effectiveness is largely dependent on the ability to distinguish between the host and pathogenic cells which are both eukaryotic, consequently determining their specificity [28]. For the treatment of *Aspergillus* infections, the three main classes of antifungal agents used are azoles, polyenes and echinocandins [29].

Azoles, in particular triazoles, are the preferred agents for the treatment and prevention of invasive aspergillosis in most patients [30]. Azoles are fungistatic agents that act by inhibiting the lanosterol C14α demethylation, thus interfering with the synthesis of ergosterol present in the fungal cell membrane. The resultant 14-α-methylsterol produced is then placed loosely within the lipid bilayers, affecting the stability of the fungal cell membrane [31]. This group of fungistatic azoles include itraconazole, voriconazole and posaconazole which are the first line for the treatment of invasive aspergillosis [30]. Even though triazole antifungals are generally well tolerated, side effects such as hepatotoxicity have been reported, with voriconazole causing life-threatening hepatitis in several patients [32,33,34]. In addition, as aspergillosis occurs mainly in immunosuppressed patients particularly cancer patients, drug interactions between triazoles and chemotherapeutic agents have also been reported. This is because triazoles and many chemotherapeutic agents are being metabolised by a common metabolic pathway, the hepatic cytochrome p450 system, thus, close monitoring is highly warranted to minimize toxicity resulting from the concurrent use of these agents [35]. Additionally, the resistance of *Aspergillus* spp. towards azoles has also been reported. A 5-year follow-up study revealed that 4.1% and 14.5% of *A. fumigatus* isolates were resistant to voriconazole and itraconazole, respectively. Among the voriconazole-resistance isolates, 95% have a cross-resistance with itraconazole. Regardless, the resistance level is highly variable depending on the type of azole drug used, the clinical settings, the patient’s background and the underlying infection [36]. In addition, the prevalence of azole-resistant *A. fumigatus* from clinical and environmental samples across Asia revealed the range of 3% in China, Japan and Kuwait to 33% in China [37]. Azole-resistant *A. fumigatus* has also been associated with a higher mortality rate [38,39]. In 2019, the U.S. Centers for Disease Control and Prevention (CDC) listed azole-resistant *A. fumigatus* on the watch list in the CDC’s antibiotic resistance threats report, a category that includes uncommon germs that potentially spread and cause significant morbidity and mortality [40].

The next group of antifungal agents are the polyenes, in which amphotericin B is used in the treatment of *Aspergillus* infections [30]. A well-known antifungal mechanism arising from this group of agents involves the interaction with ergosterol, resulting in ionic imbalance and cell death [41]. However, recent studies propose the ability of amphotericin B in forming large extramembranous aggregates that extract ergosterol from the lipid bilayers, leading to cell death [41]. Amphotericin B is usually not recommended as the first-line treatment unless the patient is contraindicated to voriconazole or it is administered as salvage therapy. Although amphotericin B is an effective agent for *Aspergillus* infection, its use is limited due to the high incidence of nephrotoxicity [14]. In 2018, widespread amphotericin B-resistance were reported in Canada among *A. fumigatus* clinical strains, which could greatly contribute to treatment failure [42].

The last group of antifungal agents used in the treatment and prophylaxis of aspergillosis are echinocandins [30]. Echinocandin antifungal agents are lipopeptides obtained from fungal secondary metabolites. Agents belonging to this class are caspofungin, micafungin and anidulafungin. They act by noncompetitively inhibiting β-1,3-glucan synthase (encoded by *fks1*) which is involved in the synthesis of β-1,3-glucan, a major polysaccharide in the cell wall of the fungi [43]. This class of agents carries the advantages of having few adverse effects and drug interactions, which are the limitations associated with the previous two groups [43]. However, among the three classes of antifungal agents, echinocandins have the least effectiveness against *Aspergillus* spp. Due to the limited oral bioavailability, echinocandins are administered only via intravenous infusion, thus are limited only to patients admitted to the hospital [44]. This contrasts with azole antifungal agents which are able to be delivered orally. Additionally, due to the limited data available on the efficacy of echinocandin monotherapy, they are used mainly in salvage therapy or when the other drugs are not preferable in the patients [30]. In 2017, the first case of echinocandin resistance in *A. fumigatus* caused by the point mutation in the *fks1* gene has been reported, causing treatment failure [45].

Interestingly, many of the current antifungals originated from natural sources. Amphotericin B, which was discovered in 1956 was derived from the bacteria *Streptomyces nodosus* [46]. On the other hand, echinocandins are semisynthetic lipopeptides obtained by chemically modifying compounds derived from natural sources. The lead compound of caspofungin, micafungin and anidulafungin were all obtained from fungi, which are *Glarea lozoyensis*, *Coleophoma empetri* and *Aspergillus nidulans,* respectively [47]. Thus, this strongly encourages the discovery of new antifungal agents via natural sources.

## 4. Anti-*Aspergillus* Activity of Plants

The development of resistance to the currently utilized antifungal drugs makes it crucial to discover new classes of antifungals from natural products including plants [48]. Various medicinal plants have demonstrated the potential to treat fungal infections, with some of them showing broad-spectrum antifungal activity [49].

Plants are a valuable source of a wide variety of biologically active chemical defence constituents, the secondary metabolites (phytoalexins and phytoanticipins) [48]. Numerous plant secondary metabolites and their derivatives have been identified as possible antimicrobial agents. Polyphenols are one of the most abundant and diverse groups of plant secondary metabolites. Their antimicrobial effects have been associated with their antioxidant properties [50]. Flavonoids, the largest group of naturally occurring phenolic compounds have been recognised for their antimicrobial activity and the structures of various flavonoid compounds with antifungal [51], antiviral [52] and antibacterial [53] properties have been extensively identified by numerous studies. Inhibition of fungal growth by flavonoids involves several underlying mechanisms, including the disruption of the plasma membrane, induction of mitochondrial dysfunction as well as inhibition of cell wall formation, cell division, RNA, protein synthesis and efflux-mediated pumping system [51]. Alkaloids are also well known for their various biological activities including anticancer, antibacterial, antidepressant, herbicidal, anti-histaminic, central nervous system stimulation, insecticidal and fungicidal activities [54]. They are heterocyclic structures containing one or more nitrogen atoms which can be classified according to their chemical structure or natural origin. The antifungal activity of alkaloids may be mediated through the disruption of mitochondrial iron-sulphur (Fe-S) cluster synthesis and cell wall integrity [55,56]. Triterpenes are proved to possess potential antifungal activity [57,58] in addition to their anticancer, anti-inflammatory, anti-oxidative and anti-viral activities [59]. Their antifungal activity may be through inhibition of mycelial growth, causing damage to the cell membrane, compromising the integrity and permeability of fungal cells, as well as by causing cation leakage from the cytoplasm [60,61,62].

Minimum inhibitory concentration (MIC) is defined as the lowest concentration of a substance that inhibits microbial growth [63]. With respect to plant extracts, many consider that only those with MIC values lower than 0.1 mg/mL in an in vitro assay are noteworthy and may be further explored for potential new antimicrobials [63]. Besides the biological activities, cytotoxicity studies of plants are of paramount importance as no crude plant extracts or natural products are considered safe for use or consumption until they are subjected to cellular toxicity and in vivo tests [64]. The higher value of 50% lethal concentration (LC_50_) in cytotoxicity tests implies that a higher quantity of the extract would be needed to cause a toxic response. Extracts with LC_50_ > 0.1 mg/mL are regarded as having negligible cytotoxicity and the American National Cancer Institute (NCI) refer to an LC_50_ of ≤0.03 mg/mL to be toxic [65,66]. Selectivity index (SI) is the ratio of toxic concentration against the effective bioactive concentration of a sample. Plant extracts with high SI values are generally expected to be much safer for human use or consumption, but this has to be confirmed with in vivo tests. SI values ≥ 10 indicate a promising hit for further investigation, and a lower SI value ≥ 3 was proposed by Mongalo et al. [65] and Peña-Morán et al. [67] as highly selective.

### 4.1. Crude Extracts

Plants are used extensively as crude materials and as sources of pure compounds. Various techniques such as Soxhlet extraction, maceration, hydro-distillation, percolation, decoction, reflux extraction, pressurized liquid extraction, supercritical fluid extraction, ultrasound assisted extraction, microwave assisted extraction, pulsed electric field extraction, and enzyme assisted extraction have been employed to extract the bioactive compounds from plant materials [68,69]. The anti-*Aspergillus* activity of plant extracts is shown in Table 1.

Twenty-five South African medicinal plants selected on the basis of their availability were investigated against three phytopathogenic fungal strains including *A. flavus* and *A. ochraceous* [60]. The acetone leaf extracts of the plants showed varying antifungal effects against *A. flavus* and *A. ochraceous*. The lowest MIC value of 0.08 mg/mL was reported for *Markhamia obtusifolia* against *A. flavus* and *Curtisia dentata* against *A. ochraceous* after 24 and 48 h of incubation, with the latter, further yielded the highest total activity of 1583 mL/g against *A. ochraceous* [60]. Gas chromatography-mass spectrometry (GC-MS) analysis of these two plants revealed the presence of triterpenoids and sterols (e.g., β-amyrin, α-amyrin, β-sitosterol) as well as vitamin E in *C. dentata* whereas neophytadiene, palmitic acid and 4-(1E)-3-hydroxy-1-propenyl)-2-methoxyphenol were the major constituents of *M. obtusifolia*. Another study by Singh et al. [70] has also reported the presence of several compounds including α-amyrin and β-sitosterol in *Acacia raddiana* heartwood extract and fractions. Benzene fractions showed the greatest effectiveness against *A. niger* (ZOI at 4 mg/disc = 9.20 mm, AI = 0.33) whereas ethyl acetate fractions exhibited the highest effectiveness against *A. flavus* (ZOI at 4 mg/disc = 10.00 mm, AI = 0.36). β-sitosterol, palmitic acid and neophytadiene from other plant species have also been reported to be effective against *Aspergillus* spp. [71,72,73].

Among the ten selected South African medicinal plants, the organic extract of *Milletia grandis* revealed the lowest MIC value of 0.01 mg/mL against *A. ochraceous* after 24 and 48 h incubation period [65]. In addition to *M. grandis*, the organic extracts of *Warburgia salutaris*, *Bauhinia galpinii*, *Harpephyllum caffrum* and *Solanum panduriforme* gave low MIC values of 0.01–0.10 mg/mL, and are thus good candidates for further investigation of their relevant antimicrobial compounds. The high phenolic contents of the plants may be responsible for their bioactivity. Furthermore, these plant species were generally non-toxic to Bovine dermis (LC_50_ = 0.01 to 0.68 mg/mL) and Vero cells (LC_50_ = 0.01 to 0.59 mg/mL), and *W. salutaris* showed the best safety margin with SI value of 34 [65].

Thirty-eight secondary metabolites of flavonoids, stilbene, phenolic acids, alkaloids and coumarin classes were identified by liquid chromatography-electrospray ionization-time-of-flight mass spectrometry (LC-ESI-TOF-MS) in the aqueous-ethanolic extract and subsequent fractions of *Zygophyllum coccineum*, an edible halophytic plant [74]. Most of the identified compounds were phenolics and flavonoids. Extracts of *Z. coccineum* demonstrated potent antifungal activity against *A. fumigatus* with MIC values of 15.63, 1.95, 3.90 and 31.25 µg/mL for its aqueous-ethanolic extract, chloroform fraction, ethyl acetate fraction and n-butanol fraction, respectively. In addition to the significant antifungal and antioxidant activity, the extract and fractions also demonstrated cytotoxic effects with IC_50_ values of 3.47, 3.19 and 2.27 µg/mL against MCF-7 (breast cancer), HCT-116 (colon cancer) and HepG2 (liver cancer) cell lines, respectively. The anticancer activity of the extract was also validated by in silico receptor-binding predicted energy levels and receptor-site docking feasibility of the major constituents found in the extract [74].

Phytochemical screening and determination of antimicrobial activities of the desert plants growing in Saudi Arabia have reported notable antifungal activity against *A. fumigatus* [58]. *Tripleurospermum auriculatum* demonstrated the greatest antifungal activity (MIC = 1.95 µg/mL, ZOI = 29.9 mm), followed by *Centarea pseudosinaica* (MIC = 1.95 µg/mL, ZOI = 28.3 mm), *Plectranthus arabicus* (MIC = 3.9 μg/mL, ZOI = 27.3 mm), *Plectranthus asirensis* (MIC = 3.9 μg/mL, ZOI = 26.4 mm), *Centarea sinaica* (MIC = 15.63 μg/mL, ZOI = 22.8 mm), *Calendula tripterocarpa* (MIC = 15.63 μg/mL, ZOI = 21.7 mm) and *Koelpinia linearis* (MIC = 62.5 μg/mL, ZOI = 17.3 mm). The high phenolic and flavonoid contents of the plants may be responsible for the protection against oxidative stress and paraneoplastic symptoms caused by cancer [58]. Furthermore, given orally to the mice, the obtained results of acute toxicity (LD_50_ up to 5000 mg/kg) and sub-chronic toxicity tests revealed that the plants have no toxicity and are considered safe for human use.

A combination of several medicinal herbs for extra therapeutic benefits is known as polyherbalism and it has been used in Ayurvedic, Chinese and Unani medicines since ancient times [75]. Multiple bioactive constituents present in the polyherbal formulations may interact with each other and result in a greater effect, a phenomenon known as synergism. With the wide therapeutic range and high efficiency of polyherbal formulations, they are used to treat various diseases including infections [76]. Among the fourteen polyherbal crude extracts obtained from different combinations of twenty-five plant species, the inhibition zone by 50 mg/mL of extracts ranged between 13.33-28.67 mm against *A. niger* and 5.00-27.00 mm against *A. fumigatus* [77]. Recipe C consists of *Foeniculum vulgare* Mill. Apiaceae, *Withania coagulans* (Stocks) Dunal. Solanaceae, *Camellia sinensis* L. Kuntze Theaceae and *Elettaria cardamomum* (L.) Maton. Zingiberaceae showed the highest antifungal activity against the tested *Aspergillus* spp. (*A. niger* and *A. fumigatus*. Moreover, its antifungal activity was also better than the standard fluconazole (ZOI = 28.67 mm for polyherbal extract C vs. 19.33 mm for fluconazole against *A. niger*; ZOI = 27.00 mm for polyherbal extract C vs. 26.67 mm for fluconazole against *A. fumigatus*). A statistically significant antifungal effect was also reported for polyherbal mixtures A, D and F against *A. niger*, *A. fumigatus* and *Fusarium graminearum* compared to fluconazole, which indicates their antifungal efficacy as fluconazole is not effective against these tested fungi [77]. The presence of essential oil in the polyherbal mixtures that consist of plants from Apiaceae and Zingiberaceae may be responsible for the antimicrobial activity. Further in vivo and clinical trials are needed to evaluate the potential of these polyherbal formulations in combating microbial resistance issues [77].

**Table 1:** Antifungal effects of plant crude extracts against *Aspergillus* spp.

Plant	Family	Extraction Solvent	Phytochemical Constituents/Main Compounds Identified	*Aspergillus* Species	Antifungal Activity						Reference
					**MGI (%)**						
*Aristolochia indica*	Aristolochiaceae	Methanol	Tannins, flavonoids, terpenoids, anthraquinone, phenolics	*A. flavus*	79						[78]
				*A. fumigatus*	75						
*Cuscuta pedicellata*	Convolvulaceae	Methanol	Tannins, flavonoids, terpenoids, phlobatannins, anthraquinone, phenolics	*A. flavus*	88						
				*A. fumigatus*	90						
*Melilotus indicus*	Fabaceae	Methanol	Tannins, flavonoids, terpenoids, anthraquinone, phenolics	*A. flavus*	63						
				*A. fumigatus*	80						
*Tribulus terrestris*	Zygophyllaceae	Methanol	Flavonoids, terpenoids, phlobatannins, anthraquinone, phenolics	*A. flavus*	83 (fruit), 79 (leaf)						
				*A. fumigatus*	87 (fruit), 85 (leaf)						
					**MIC (mg/mL)**						
*Colebrookia oppositifolia*	Lamiaceae	Water	Terpenoids, saponins, tannins, sugars, phenolics, flavonoids, cardiac glycosides	*A. flavus*	149						[79]
					**ZOI at 100 μg/disc (mm)**						
*Rhus Punjabensis* Stewart	Anacardiaceae	n-hexane	Phenolics, flavonoids	*A. fumigatus*	n.a.						[80]
				*A. niger*	7 (stem)						
				*A. flavus*	n.a.						
		Chloroform	Phenolics, flavonoids	*A. fumigatus*	n.a.						
				*A. niger*	n.a.						
				*A. flavus*	n.a.						
		Acetone	Phenolics, flavonoids	*A. fumigatus*	13 (leaf)						
				*A. niger*	n.a.						
				*A. flavus*	n.a.						
		Ethyl acetate	Phenolics (gallic acid), flavonoids	*A. fumigatus*	7.5 (leaf)						
				*A. niger*	n.a.						
				*A. flavus*	11 (stem)						
		Ethanol	Phenolics (rutin), flavonoids	*A. fumigatus*	n.a.						
				*A. niger*	n.a.						
				*A. flavus*	9 (leaf)						
		Methanol	Phenolics (gallic acid, rutin), flavonoids	*A. fumigatus*	n.a.						
				*A. niger*	n.a.						
				*A. flavus*	14 (leaf)						
		Ethanol and chloroform	Phenolics (catechin, gallic acid, apigenin), flavonoids	*A. fumigatus*	n.a.						
				*A. niger*	n.a.						
				*A. flavus*	11.2 (leaf), 9.5 (stem)						
		Methanol and chloroform	Phenolics (gallic acid, rutin, apigenin), flavonoids	*A. fumigatus*	12 (leaf)						
				*A. niger*	n.a.						
				*A. flavus*	n.a.						
		Acetone and ethyl acetate	Phenolics, flavonoids	*A. fumigatus*	n.a.						
				*A. niger*	n.a.						
				*A. flavus*	11.5 (leaf), 9 (stem)						
		Ethanol and ethyl acetate	Phenolics, flavonoids	*A. fumigatus*	n.a.						
				*A. niger*	n.a.						
				*A. flavus*	10 (leaf)						
		Methanol and ethyl acetate	Phenolics, flavonoids	*A. fumigatus*	n.a.						
				*A. niger*	n.a.						
				*A. flavus*	11 (leaf)						
					**GI at 7.5 mg/mL (%)**						
*Astragalus eremophilus*	Fabaceae	Methanol (70%)	Phenolics, tannins, saponins, flavonoids	*A. flavus*	63.1						[81]
				*A. fumigatus*	61.3						
				*A. niger*	57.1						
*Melilotus indicus* (L.)	Fabaceae	Methanol (70%)	Phenolics, tannins, saponins, flavonoids	*A. fumigatus*	70.1						
				*A. flavus*	67.5						
				*A. niger*	64						
					**MIC (mg/mL)**						
*Bauhinia galpinii*	Fabaceae	Water	Flavonoids, phenolics	*A. parasiticus*	0.39						[65]
				*A. ochraceous*	1.3						
				*A. flavus*	1.56						
		Methanol and dichloromethane		*A. parasiticus*	**0.1**						
				*A. ochraceous*	0.2						
				*A. flavus*	**0.02**						
*Carpobrotus eludis*	Aizoaceae	Water	Flavonoids, phenolics	*A. parasiticus*	0.65						
				*A. ochraceous*	1.56						
				*A. flavus*	6.25						
		Methanol and dichloromethane		*A. parasiticus*	**0.01**						
				*A. ochraceous*	**0.1**						
				*A. flavus*	0.2						
*Harperphylum caffrum*	Anacardiaceae	Water	Flavonoids, phenolics	*A. parasiticus*	0.65						
				*A. ochraceous*	1.3						
				*A. flavus*	2.6						
		Methanol and dichloromethane		*A. parasiticus*	**0.04**						
				*A. ochraceous*	**0.02**						
				*A. flavus*	2.6						
*Milletia grandis*	Fabaceae	Water	Flavonoids, phenolics	*A. parasiticus*	1.17						
				*A. ochraceous*	1.3						
				*A. flavus*	1.56						
		Methanol and dichloromethane		*A. parasiticus*	**0.01**						
				*A. ochraceous*	**0.01**						
				*A. flavus*	**0.1**						
*Solanum aculeastrum*	Solanaceae	Water	Flavonoids, phenolics	*A. parasiticus*	0.2						
				*A. ochraceous*	0.39						
				*A. flavus*	0.39						
		Methanol and dichloromethane		*A. parasiticus*	0.2						
				*A. ochraceous*	0.2						
				*A. flavus*	0.2						
*Solanum panduriforme*	Solanaceae	Water	Flavonoids, phenolics	*A. parasiticus*	0.39						
				*A. ochraceous*	0.78						
				*A. flavus*	0.78						
		Methanol and dichloromethane		*A. parasiticus*	**0.02**						
				*A. ochraceous*	**0.02**						
				*A. flavus*	0.2						
*Ziziphus mucronata*	Rhamnaceae	Water	Flavonoids, phenolics	*A. parasiticus*	3.13						
				*A. ochraceous*	1.3						
				*A. flavus*	1.56						
		Methanol and dichloromethane		*A. parasiticus*	0.2						
				*A. ochraceous*	**0.1**						
				*A. flavus*	0.4						
					**MIC (μg/mL)**			**ZOI (mm)**			
*Calendula tripterocarpa*	Asteraceae	Ethanol (95%)	Sterols and/or triterpenes, carbohydrates and/or glycosides, flavonoids, tannins, anthraquinones, alkaloids and/or nitrogenous bases, protein and/or amino acids	*A. fumigatus*	**15.63**			21.7			[58]
*Centarea sinaica*	Asteraceae	Ethanol (95%)	Sterols and/or triterpenes, carbohydrates and/or glycosides, flavonoids, tannins, anthraquinones, alkaloids and/or nitrogenous bases, protein and/or amino acids	*A. fumigatus*	**15.63**			22.8			
*Centarea pseudosinaica*	Asteraceae	Ethanol (95%)	Sterols and/or triterpenes, carbohydrates and/or glycosides, flavonoids, tannins, anthraquinones, alkaloids and/or nitrogenous bases, protein and/or amino acids	*A. fumigatus*	**1.95**			28.3			
*Koelpinia linearis*	Asteraceae	Ethanol (95%)	Sterols and/or triterpenes, carbohydrates and/or glycosides, flavonoids, tannins, anthraquinones, alkaloids and/or nitrogenous bases, protein and/or amino acids	*A. fumigatus*	**62.5**			17.3			
*Plectranthus arabicus*	Lamiaceae	Ethanol (95%)	Sterols and/or triterpenes, carbohydrates and/or glycosides, flavonoids, tannins, anthraquinones, alkaloids and/or nitrogenous bases, protein and/or amino acids	*A. fumigatus*	**3.9**			27.3			
*Plectranthus asirensis*	Lamiaceae	Ethanol (95%)	Sterols and/or triterpenes, carbohydrates and/or glycosides, flavonoids, tannins, anthraquinones, alkaloids and/or nitrogenous, bases, protein and/or amino acids	*A. fumigatus*	**3.9**			26.4			
*Tripleurospermum auriculatum*	Asteraceae	Ethanol (95%)	Sterols and/or triterpenes, carbohydrates and/or glycosides, flavonoids, tannins, anthraquinones, alkaloids and/or nitrogenous bases, protein and/or amino acids	*A. fumigatus*	**1.95**			29.9			
					**MIC (μg/mL)**						
*Hypericum hircinum* subsp. *majus*	Hypericaceae	Methanol	Phenolics (benzoates and cinnamates, ellagic acid, chlorogenic acid, neochlorogenic acid, flavonols, hyperoside, quercetin-3-glucoside, quercetin-3-glucuronide, quercetin-3-sulfate, flavan-3-ols, catechin, epicatechin, procyanidin B2)	*A. glaucus*	>500						[82]
		Ethanol (80%)			>500						
		Water (infusion)			250						
					**ZOI at 50 μg/disc (mm)**						
*Silybum marianum*	Asteraceae	Ethanol	Tannins, glycosides, terpenoids, alkaloids, flavonoids	*A. oryzae*	7.44						[83]
*Thymus daenensis*	Lamiaceae	Ethanol	Tannins, glycosides, terpenoids, alkaloids, flavonoids	*A. oryzae*	10.64						
					**MIC (mg/mL)**		**MGI at MIC (%)**		**Total activity (mL/g)**		
*Curtisia dentata*	Cornaceae	Acetone (30%)	Vitamin E, γ-sitosterol, α-amyrin, β-amyrin, 24-methyl-9,19-cyclolanost-25-en-3-ol	*A. flavus*	0.63		8.35		2010		[60]
				*A. ochraceous*	**0.08**		14.2		**1583**		
*Markhamia obtusifolia*	Bignoniaceae	Acetone (30%)	4-((1E)-3-hydroxy-1-propenyl)-2-methoxyphenol (coniferol), neophytadiene, palmitic acid	*A. flavus*	**0.08**		18.38		958		
				*A. ochraceous*	0.16		20.4		479		
					**GI at 150 μL (%)**						
*Adiantum incisum*	Pteridaceae	WaterMethanoln-hexane	Alkaloids, cardiac glycosides, coumarins, flavonoids, glycosides, phenols, phlobatannins, saponins, steroids, tannins, terpenoids	*A. niger*	50.73-78.3						[84]
				*A. flavus*	n.a.						
					**MIC (μg/mL)**						
*Aquilaria sinensis*	Thymelaeaceae	Ethanol (95%)	Sesquiterpenes, 2-(2-phenylethyl) chromone derivatives	*A. niger*	**63**-590						[85]
					**MIC (mg/mL)**						
*Catharanthus roseus* (L.) G. Don	Apocynaceae	Methanol	Saponins, phenolics (gallic acid, apigenin, kaempferol)	*A. niger*	>10						[86]
					**ZOI (mm)**						
*Berberis aristate* DC	Berberidaceae	Methanol	Carbohydrates, alkaloids, phenolics, glycosides, acidic compounds, proteins and amino acids, flavonoids, resins, sterols	*A. terreus*	14.2 (3000 μg/mL)						[87]
					12.9 (1500 μg/mL)						
					11.7 (750 μg/mL)						
					10.3 (300 μg/mL)						
					**MIC (μg/mL)**						
*Gentiana crassicaulis* Duthie ex Burkill	Gentianaceae	Ethanol	Bisphosphocholines (irlbacholine, gentianalines A, B and C)	*A. fumigatus*	**9.99/12.5**						[88]
		Chloroform			**44.3/100**						
					**MIC (μg/mL)**		**MFC (μg/mL)**		**IZD at 50 mg/mL (mm)**		
*Lycium shawii* Roem. & Schult.	Solanaceae	Water	Phenolics, flavonoids, alkaloids, tannins, glycosides, terpenoids, steroids	*A. niger*	**32**		64		10.6		[89]
		Methanol			**4**		16		54		
		Ethanol			**16**		16		34.1		
		Ethyl acetate			**16**		32		39.5		
					**MIC (μg/mL)**						
*Moringa oleifera*	Moringaceae	Ethanol (50%)	Alkaloids, tannins, flavonoids, steroids, saponins, polyphenols, glycosides, carbohydrates, proteins, amino acids	*A. niger*	**62.5**						[90,91]
				*A. flavus*	**62.5**						
					**ZOI at 0.3 mL (mm)**						
*Trigonella foenum-graecum* L.	Fabaceae	Purified water and ethanol (10%)	Saponins, steroidal saponins, flavonoids, phenols, proteins (aqueous and ethanolic extracts) carbohydrates, alkaloids (aqueous extract)	*A. niger*	29						[92]
		Ethanol			30						
					**MIC (mg/mL)**			**MFC (mg/mL)**			
*Anabasis articulata*	Amaranthaceae	Ethanol (95%)	Apigenin-7-O-glucoside (apigetrin), apigenin-7-O-glucoside (apigetrin), chlorogenic acid, hyperoside, quercetin	*A. niger*	12.5			25			[93]
*Rumex vesicarius*	Polygonaceae	Ethanol (95%)	Apigenin-7-O-glucoside (apigetrin), apigenin-7-O-glucoside (apigetrin), chlorogenic acid, hyperoside, quercetin	*A. niger*	50			100			
					**MIC (mg/mL)**						
*Avicennia marina*	Acanthaceae	Ethanol (40%) and sequential separation with petroleum ether, ethyl acetate, ethanol, chloroform and water	1,2-benzenedicarboxylic acid, cis-cinnamic acid, hexadecanoic acid, 2,6,10,14,18,22-tetracosahexae, 25-ethyl-27-norcholesta-5,24(Z), 1-tetradecene, taraxasterol, hydroxymethylfurfural, 1-deoxy-D-altritol	*A. fumigatus*	0.25 (ethanol (40%))						[94]
					n.a. (ethyl acetate, petroleum ether, chloroform, water)						
					**MIC (mg/mL)**			**MFC (mg/mL)**			
*Ilex paraguariensis*	Aquifoliaceae	Water	Caffeoylglucose I, caffeoylglucose IV, 6-caffeoylglucose, 4-p-coumaroylquinic acid, neochlorogenic acid, chlorogenic acid, cryptochlorogenic acid, 3,4-dicaffeoylquinic acid, 3,5-dicaffeoylquinic acid, 4,5-dicaffeoylquinic acid, 3-ferruoylquinic acid, 4- ferruoylquinic acid, 5- ferruoylquinic acid, rutin, quercetin-glucoside, kaempferol-3-O-rutinoside, isorhamnetin-3-O-rutinoside, isorhamnetin-3-O-glucoside, isorhamnetin 3-O-acetylglucoside	*A. niger*	2.06			>2.06			[95]
*Ilex aquifolium* L.	Aquifoliaceae	Water	3-p-coumaroylquinic acid, 4-p-coumaroylquinic acid, neochlorogenic acid, chlorogenic acid, cryptochlorogenic acid, 3,4-dicaffeoylquinic acid, 3,5-dicaffeoylquinic acid, 4,5-dicaffeoylquinic acid, 3-ferruoylquinic acid, 4- ferruoylquinic acid, 5- ferruoylquinic acid, quercetin-pentoside-hexoside, quercetin-7-O-rutinoside, rutin, quercetin-glucoside, kaempferol-3-O-rutinoside	*A. niger*	2.06			>2.06			
*Ilex aquifolium* ‘Argentea Marginata’	Aquifoliaceae	Water	3-p-coumaroylquinic acid, 4-p-coumaroylquinic acid, neochlorogenic acid, chlorogenic acid, cryptochlorogenic acid, 3,4-dicaffeoylquinic acid, 3,5-dicaffeoylquinic acid, 4,5-dicaffeoylquinic acid, 3-ferruoylquinic acid, 4- ferruoylquinic acid, 5- ferruoylquinic acid, quercetin-pentoside-hexoside, quercetin-7-O-rutinoside, rutin, quercetin-glucoside, kaempferol-3-O-rutinoside	*A. niger*	2.06			>2.06			
*Ilex × meserveae* ‘Blue Angel’	Aquifoliaceae	Water	Neochlorogenic acid, chlorogenic acid, cryptochlorogenic acid, 4,5-dicaffeoylquinic acid, 4- ferruoylquinic acid, 5- ferruoylquinic acid, quercetin-di-glucoside, quercetin-deoxyhexoside-hexoside, flavonoid-derivate, kaempferol-rutinoside-hexoside, isorhamnetin-3-O-gentiobioside, isorhamnetin-rutinoside-glucoside, isorhamnetin derivate, myricetin-hexoside, quercetin-pentoside-hexoside, quercetin-7-O-rutinoside, rutin, quercetin-glucoside, kaempferol-3-O-rutinoside, isorhamnetin-3-O-rutinoside, isorhamnetin-3-O-glucoside, isorhamnetin 3-O-acetylglucoside	*A. niger*	2.06			>2.06			
					**GI at 20 mg/mL (%)**						
*Otostegia limbata* (Benth.) Boiss.	Lamiaceae	Methanol	Anthocyanin, flavonoids, phenols, isoflavones	*A. terreus*	68						[96]
					**ZOI at 100 μL (mm)**			**AI (%)**			
*Withania coagulans*	Solanaceae	Methanol and chloroform	Withaferin, withanolide	*A. niger*	25			75			[97]
					**MIC (μg/mL)**						
*Zygophyllum coccineum* L.	Zygophyllaceae	Aqueous ethanol (70%), fractioned in chloroform, ethyl acetate and n-butanol	Isorhamnetin-3-O-glucoside, zygophyloside-G zygophyloside-F,	*A. fumigatus*	**15.63**						[74]
					**1.95**						
					**3.9**						
					**31.25**						
					**ZOI at 4 mg/disc (mm)**			**AI**			
*Acacia raddiana* Willd	Leguminosae	Ethanol	Octacosanol, monacosanol, β-sitosterol octacosanoate, β-sitosterol acetate, α-amyrin, β-sitosterol, betulin friedelin, D-pinitol	*A. niger*	n.a.			n.a.			[70]
				*A. flavus*	n.a.			n.a.			
		Petroleum ether	Octacosanol, monacosanol, β-sitosterol octacosanoate, β-sitosterol acetate, α-amyrin, β-sitosterol, betulin friedelin, D-pinitol	*A. niger*	8			0.3			
				*A. flavus*	8.68			0.32			
		Benzene	Octacosanol, monacosanol, β-sitosterol octacosanoate, β-sitosterol acetate, α-amyrin, β-sitosterol, betulin friedelin, D-pinitol	*A. niger*	9.2			0.33			
				*A. flavus*	9.25			0.34			
		Ethyl acetate	Octacosanol, monacosanol, β-sitosterol octacosanoate, β-sitosterol acetate, α-amyrin, β-sitosterol, betulin friedelin, D-pinitol	*A. niger*	8.74			0.32			
				*A. flavus*	10			0.36			
					**MIC (mg/mL)**						
*Alchornea laxiflora*	Euphorbiaceae	Acetone	Phenolics, flavonoids	*A. fumigatus*	1.25						[66]
				*A. flavus*	0.46						
		Methanol		*A. fumigatus*	1.25						
				*A. flavus*	>2.50						
		Ethanol		*A. fumigatus*	1.56						
				*A. flavus*	2.5						
		Cold water		*A. fumigatus*	0.31						
				*A. flavus*	>2.50						
		Hot water		*A. fumigatus*	1.25						
				*A. flavus*	>2.50						
*Ficus exasperata*	Moraceae	Acetone	Phenolics, flavonoids	*A. fumigatus*	0.46						
				*A. flavus*	0.23						
		Methanol		*A. fumigatus*	0.93						
				*A. flavus*	2.5						
		Ethanol		*A. fumigatus*	1.25						
				*A. flavus*	0.51						
		Cold water		*A. fumigatus*	0.62						
				*A. flavus*	0.15						
		Hot water		*A. fumigatus*	0.62						
				*A. flavus*	0.38						
*Morinda lucida*	Rubiaceae	Acetone	Phenolics, flavonoids	*A. fumigatus*	1.56						
				*A. flavus*	0.15						
		Methanol		*A. fumigatus*	1.25						
				*A. flavus*	2.5						
		Ethanol		*A. fumigatus*	1.25						
				*A. flavus*	2.5						
		Cold water		*A. fumigatus*	0.15						
				*A. flavus*	**0.03**						
		Hot water		*A. fumigatus*	2.5						
				*A. flavus*	0.83						
*Jatropha gossypiifolia*	Euphorbiaceae	Acetone	Phenolics, flavonoids	*A. fumigatus*	0.31						
				*A. flavus*	0.62						
		Methanol		*A. fumigatus*	0.62						
				*A. flavus*	0.83						
		Ethanol		*A. fumigatus*	0.62						
				*A. flavus*	0.62						
		Cold water		*A. fumigatus*	0.31						
				*A. flavus*	0.15						
		Hot water		*A. fumigatus*	>2.50						
				*A. flavus*	0.51						
*Ocimum gratissimum*	Lamiaceae	Acetone	Phenolics, flavonoids	*A. fumigatus*	2.5						
				*A. flavus*	**0.03**						
		Methanol		*A. fumigatus*	1.25						
				*A. flavus*	1.66						
		Ethanol		*A. fumigatus*	2.5						
				*A. flavus*	1.66						
		Cold water		*A. fumigatus*	0.62						
				*A. flavus*	0.31						
		Hot water		*A. fumigatus*	>2.50						
				*A. flavus*	>2.50						
*Acalypha wilkesiana*	Euphorbiaceae	Acetone	Phenolics, flavonoids	*A. fumigatus*	**0.07**						
				*A. flavus*	0.62						
		Methanol		*A. fumigatus*	2.5						
				*A. flavus*	>2.50						
		Ethanol		*A. fumigatus*	0.15						
				*A. flavus*	0.15						
		Cold water		*A. fumigatus*	2.5						
				*A. flavus*	>2.50						
		Hot water		*A. fumigatus*	>2.50						
				*A. flavus*	0.46						
					**MIC (μg/mL)**			**ZOI (mm)**			
*Zea mays* L.	Poaceae	n-Hexane	Phenolics, flavonoids, alkaloids, masonic acid, saponins	*A. niger*	n.a.			n.a.			
		Chloroform			n.a.			n.a.			
		Ethyl acetate			365			10.22			
		n-Butanol			260			13.45			
		Methanol			200			15.25			

The bold value for antifungal activity indicates noteworthy activity (MIC ≤ 0.1 mg/mL). “-”: not determined; AI: activity index; GI: growth inhibition; MFC: minimum fungicidal concentration; MGI: mycelia growth inhibition; MIC: minimum inhibitory concentration; n.a.: not active; ZOI: zone of inhibition.

### 4.2. Essential Oils

Essential oils (EOs) are mixtures of volatile compounds obtained from the plants by hydro-distillation, steam distillation, dry distillation, or mechanical cold pressing of plants [98]. They are mainly composed of terpenes, terpenoids and phenylpropanoids with other compounds such as fatty acids, oxides and sulphur derivatives that may also be found in EOs [98]. Many EOs have been reported to exhibit antimicrobial properties which makes them very important in the pharmaceutical, agronomic, food, sanitary, cosmetic and perfume industries [98,99]. Table 2 shows the antifungal effects of EOs against *Aspergillus* spp.

EOs obtained by hydro-distillation (HD) and microwave-assisted extraction (MAE) methods from *Thymus decussatus* were tested against several fungi including *A. niger* [100]. Both HD and MAE samples of *T. decussatus* showed strong antifungal activity against *A. niger*, with an MIC value of 0.49 μg/mL and inhibition zone of 47 mm (HD) and 48 mm (MAE). GC-MS analysis revealed the presence of oxygenated monoterpenes as the main components of both EOs with carvacrol as the main compound (94.40% for HD and 75.91% for MAE). The strong antimicrobial activity of *T. decussatus* may also be attributed to another main compound, p-cymene (3.61% for HD and 16.98% for MAE), in addition to carvacrol which has been reported to show antimicrobial activity in past studies. EOs from *Asteriscus graveolens* have been reported to show fungicidal activity against *Alternaria* sp. and *Penicillium expansum* in previous studies [101] and a recent study by Aljeldah [102] showed that the *A. graveolens* EO also demonstrated a good antifungal effect against *Candida albicans*, *A. niger*, *A. flavus* and *Fusarium oxysporum*. The MIC results showed that *A. graveolens* EO strongly inhibited *Aspergillus* spp. used for testing, recording MICs values of 24.50 μg/mL against *A. niger* and 23.74 μg/mL against *A. flavus*. The higher amounts of α-thujone (17.92%) and carvacrol (14.14%) revealed by GC-MS may be responsible for the antifungal activity of the EO.

EO from the fresh leaves of *Murraya paniculata* showed moderate antifungal to strong antifungal activity against *A. niger* (MIC = 0.2 mg/mL), *A. fumigatus* (MIC = 0.1 mg/mL) and *A. parasiticum* (MIC = 0.2 mg/mL) [103]. Thirteen compounds, with β-caryophyllene (57.57%) as the major compound, were identified in the EO. The antifungal action of *M. paniculata* EO is due to the synergistic action among its components, as implied by higher MIC and MFC values of β-caryophyllene compared with the EO. Since the ratios of the lethal concentration to the minimum inhibitory concentration (MFC/MIC) were all less than or equal to 4, the EO oil and β-caryophyllene demonstrated fungicidal action instead of fungistatic action. From the time–kill curve study, the EO showed a good and rapid dose-dependent fungicidal effect within 4-8 hours for *A. niger*. Furthermore, the SI value of 3.06 was obtained when testing against tumorous cells of hepatocytes and fibroblasts. The toxicity of EOs against fungi may be attributed to terpenes and phenolic compounds, with several underlying mechanisms such as disruption of cell membranes, causing leakage of cellular material, inhibition of mitochondrial ATPase and the electron transport chain [104].

The antifungal activities of the EOs are not distinguished against all strains and their effective time of action is limited by their high volatility. Furthermore, their features such as hydrophobicity, instability and possible toxicity may compromise their use [98]. Encapsulation of the EOs may help to overcome these limitations [105,106]. Several techniques have been employed to encapsulate EOs, and emulsified solution is commonly used as a basis for encapsulation. Physical encapsulating methods include extrusion, fluidization, lyophilization, solvent removal, spray dryer, supercritical fluid technology, whereas coacervation, ionic gelation, liposomes and miniemulsion polymerization are the chemical methods used to obtain microcapsules of EOs [106]. Substances used as wall materials for microencapsulation of EOs include gums (e.g., arabic, almond, sodium alginate), proteins (e.g., whey, soy, casein, gelatin), polysaccharides (e.g., starch, dextrin, maltodextrin, cyclodextrin, chitosan, cellulose) and lipids (e.g., waxes, paraffin, fats).

**Table 2:** Antifungal effects of essential oils against *Aspergillus* spp.

Essential Oil	Extraction Method	Phytochemical Constituents/ Main Compounds Identified	*Aspergillus* Species	Antifungal Activity		Reference
				**ZOI at 75% (mm)**		
*Artemisia abrotanum*	Clevenger hydro-distillation	Terpenes, terpenoids, aromatic, aliphatic constituents	*A. flavus*	11.89		[107]
*Cinnamomum* *zylanicum*	Clevenger hydro-distillation	Terpenes, terpenoids, aromatic, aliphatic constituents	*A. flavus*	n.a.		
*Clove eugenia* *caryophyllus*	Clevenger hydro-distillation	Terpenes, terpenoids, aromatic, aliphatic constituents	*A. flavus*	28.14		
*Eucalyptus* *camaldulensis*	Clevenger hydro-distillation	Terpenes, terpenoids, aromatic, aliphatic constituents	*A. flavus*	16.56		
*Marjoram majorana hortensis*	Clevenger hydro-distillation	Terpenes, terpenoids, aromatic, aliphatic constituents	*A. flavus*	11.89		
				**MGI at 8 μL/mL (%)**		
*Curcuma longa* L.	Clevenger hydro-distillation	Ar-tumerone, tumerone, β-sesquiphellandrene, curcumene	*A. flavus*	93.41		[108]
				**GI at 1500 μL/L (%)**		
*Eucalyptus* sp.	Clevenger steam distillation	1,8-cineol	*A. tubingensis*	60.88 48.44		[109]
*Heracleum persicum*	Clevenger steam distillation	Hexyl ester, n-octyl acetate, pulegone, octyl ester	*A. flavus*	n.a. n.a.		
*Zhumeria majdae*	Clevenger steam distillation	Linalool, camphor	*A. flavus* *A. tubingensis*	25.77 64.4		
				**MIC (μg/mL)**	**ZOI (mm)**	
*Ferulago trifida* Boiss	Clevenger hydro-distillation	Germacrene D, caryophyllene oxide	*A. niger*	n.a.	n.a.	[110]
				**MIC (μL/mL)**	**MFC (μL/mL)**	
*Luvunga scandens* Roxb.	Hydro-distillation	Phenolics	*A. flavus*	3.5	>5.0	[111]
				**MIC (μL/mL)**		
*Mentha cardiaca* L.	Clevenger hydro-distillation	β-myrcene, limonene, 1,8-cineole, cis-dihydrocarvone, carvone, β-bourbonene	*A. flavus*	1.25		[112]
				**MIC (mg/mL)**	**MFC (mg/mL)**	
*Murraya paniculata*	Clevenger hydro-distillation	β-caryophyllene, α-caryophyllene, α-zingiberene	*A. niger* *A. fumigatus* *A. parasiticum*	0.2 **0.1** 0.2	0.2 0.5 0.5	[103]
				**MIC (μg/mL)**		
*Abies balsamea*	-	β-pinene, δ-3-carene, α-pinene, sylvestrene, bornyl acetate, camphene	*A. niger*	1250		[113]
*Abies sibirica*	-	Camphene, bornyl acetate, α-pinene, δ-3-carene, limonene	*A. niger*	625		
*Anthemis nobilis*	-	α-pinene, isobutyl angelate, methallyl angelate, 3-methylpentyl angelate	*A. niger*	625		
*Betula lenta*	-	Methyl salicylate	*A. niger*	625		
*Boswellia carteri*	-	Limonene, β-caryophyllene, p-cymene, δ-cadinene, α-copaene	*A. niger*	625		
*Cananga odorata*	-	Germacrene D, β-caryophyllene, (E, E)-α-farnesene, benzyl benzoate, geranyl acetate	*A. niger*	1250		
*Cinnamomum cassia*	-	(E)-cinnamaldehyde, (E)-o-methoxycinnamaldehyde	*A. niger*	**78**		
*Cinnamomum zeylanicum*	-	(E)-cinnamaldehyde, eugenol, (E)-cinnamyl acetate	*A. niger*	**78**		
*Cistus ladanifer*	-	α-pinene, viridiflorene, bornyl acetate, viridoflorol	*A. niger*	625		
*Citrus aurantifolia*	-	Limonene, β-pinene, γ-terpinene	*A. niger*	625		
*Citrus aurantium*	-	Linalyl acetate, linalool	*A. niger*	625		
*Citrus bergamia*	-	Limonene, linalyl acetate, linalool, γ-terpinene, β-pinene	*A. niger*	625		
*Citrus limon*	-	Limonene, β-pinene, γ-terpinene	*A. niger*	625		
*Citrus reticulata*	-	Limonene	*A. niger*	625		
*Citrus sinensis*	-	Limonene	*A. niger*	625		
*Citrus × paradisi*	-	Limonene	*A. niger*	313		
*Commiphora myrrha*	-	Furanoeudesma-1,3-diene, curzerene, lindestrene, α-pinene, neryl acetate	*A. niger*	625		
*Copaifera officinalis*	-	β-caryophyllene	*A. niger*	1250		
*Copaifera spp.*	-	β-caryophyllene, trans-α-bergamotene, α-copaene, α-humulene	*A. niger*	625		
*Coriandrum sativum*	-	Linalool, (2E)-decenal, (2E)-decen-1-ol, n-decanal	*A. niger*	313		
*Coriandrum sativum*	-	Linalool, α-pinene	*A. niger*	625		
*Cupressus* *sempervirens*	-	α-pinene, δ-3-carene	*A. niger*	1250		
*Cymbopogon flexuosus*	-	Geranial, neral, geraniol, geranyl acetate	*A. niger*	313		
*Elettaria cardamomum*	-	α-terpinyl acetate, 1,8-cineole, linalyl acetate	*A. niger*	625		
*Eucalyptus radiata*	-	1,8-cineole, α-terpineol	*A. niger*	313		
*Eugenia caryophyllata*	-	Eugenol, eugenyl acetate, β-caryophyllene	*A. niger*	156		
*Foeniculum vulgare*	-	(E)-anethole, limonene, fenchone	*A. niger*	625		
*Gualtheria* *fragrantissima*	-	Methyl salicylate	*A. niger*	625		
*Helichrysum italicum*	-	Neryl acetate, α-pinene, γ-curcumene, β-selinene, β-caryophyllene, italicene, valencene	*A. niger*	1250		
*Helichrysum italicum*	-	Neryl acetate, γ-curcumene, α-pinene	*A. niger*	625		
*Juniperus communis*	-	α-pinene, myrcene, sabinene, β-pinene, β-caryophyllene	*A. niger*	625		
*Juniperus virginiana*	-	α-cedrene, cis-thujopsene, cedrol, β-cedrene	*A. niger*	625		
*Lavandula angustifolia*	-	Linalyl acetate, linalool	*A. niger*	625		
*Melaleuca alternifolia*	-	Terpinen-4-ol, γ-terpinene, α-terpinene	*A. niger*	625		
*Melissa officinalis*	-	Geranial, neral, β-caryophyllene	*A. niger*	313		
*Mentha piperita*	-	Menthol, menthone, menthyl acetate, 1,8-cineole	*A. niger*	625		
*Mentha spicata*	-	Carvone, limonene	*A. niger*	313		
*Myristica fragrans*	-	Sabinene, myristicin, α-pinene, β-pinene, sylvestrene	*A. niger*	625		
*Myrtis communis*	-	α-pinene, 1,8-cineole, limonene	*A. niger*	1250		
*Nardostachys* *jatamansi*	-	Viridiflorene, 6,9-guaiadiene, valeranone, nardosina-7,9,11-triene, β-gurjunene, valerana-7,11-diene, nardol	*A. niger*	625		
*Nepeta cataria*	-	4aα,7α,7aβ-nepetalactone, 4aα,7α, 7aα- nepetalactone, β-caryophyllene	*A. niger*	313		
*Ocimum basilicum*	-	Linalool, 1,8-cineole, trans-α-bergamotene	*A. niger*	313		
*Origanum majorana*	-	Terpinen-4-ol, γ-terpinene, trans- sabinene hydrate, α-terpinene, sabinene	*A. niger*	625		
*Origanum vulgare*	-	Carvacrol, γ-terpinene	*A. niger*	156		
*Pelargonium* *graveolens*	-	Citronellol, iso-menthone, geraniol	*A. niger*	625		
*Picea mariana*	-	Bornyl acetate, camphene, α-pinene, δ-3-carene	*A. niger*	625		
*Piper nigrum*	-	β-caryophyllene, limonene, β-pinene, sabinene, α-pinene, δ-3-carene	*A. niger*	625		
*Pogostemon cablin*	-	Patchouli alcohol, α-bulnesene, α-guaiene, seychellene, α-patchoulene	*A. niger*	156		
*Pseudotsuga menziesii*	-	β-pinene, sabinene, terpinolene, δ-3-carene, α-pinene	*A. niger*	625		
*Rosmarinus officinalis*	-	1,8-cineole, α-pinene, camphor, β-pinene	*A. niger*	625		
*Salvia officinalis*	-	Cis-thujone, camphor, 1,8-cineole, camphene, α-pinene	*A. niger*	1250		
*Salvia sclarea*	-	Linalyl acetate	*A. niger*	1250		
*Santalum album*	-	(Z)-α-santalol, (Z)-β-santalol, (Z)-α-trans-bergamotol	*A. niger*	313		
*Santalum austrocaledonicum*	-	(Z)-α-santalol, (Z)-β-santalol, (Z)-lanceol	*A. niger*	313		
*Santalum paniculatum*	-	(Z)-α-santalol, (Z)-β-santalol, (Z)-lanceol, (Z)-α-trans-bergamotol	*A. niger*	156		
*Tanacetum annuum*	-	Sabinene, myrcene, camphor, α-phellandrene, p-cymene, chamazulene	*A. niger*	625		
*Thuja plicata*	-	Methyl thujate, methyl myrtenate	*A. niger*	313		
*Thymus vulgaris*	-	Thymol, carvacrol, p-cymene, β-caryophyllene, γ-terpinene	*A. niger*	156		
*Vetiveria zizanoides*	-	(E)-isovalencenol, khusimol, α-vetivone	*A. niger*	**78**		
*Zingiber officinale*	-	α-zingiberene, camphene, β-sesquiphellandrene, ar-curcumene, β-phellandrene, β-bisabolene	*A. niger*	625		
				**MIC (mg/mL)**		
*Salvia dolomitica*	Hydro-distillation	1,8-cineole, β-caryophyllene	*A. niger* *A. flavus*	8.13 16.26		[114]
*Salvia somalensis*	Hydro-distillation	Camphor, bornyl acetate, δ-cadinene	*A. niger* *A. flavus*	8.52 17.04		
				**MIC (μL/mL)**		
*Illicium verum* Hook.f.	Clevenger hydro-distillation	Estragole, anethole	*A. flavus*	0.7		[115]
				**MGI (%)**		
*Solidago canadensis* L.	Clevenger hydro-distillation	α-pinene, limonene, bornyl acetate, β-elemene, germacrene D	*A. niger*	15		[116]
				**MIC (μg/mL)**		
*Eupatorium serotinum* Michx.	Hydro-distillation using a Likens-Nickerson apparatus with continuous extraction with dichloromethane	Germacrene D, palustrol, cyclocolorenone	*A. niger*	313		[117]
*Eurybia macrophylla* (L.) Cass.	Hydro-distillation using a Likens-Nickerson apparatus with continuous extraction with dichloromethane	β-pinene, limonene, terpinolene, germacrene D, germacrene B	*A. niger*	625		
*Eutrochium* *purpureum* (L.) E.E. Lamont	Hydro-distillation using a Likens-Nickerson apparatus with continuous extraction with dichloromethane	Hexanal, (2E)-hexenal, methyl salicylate, eugenol	*A. niger*	625		
*Polymnia* *canadensis* L.	Hydro-distillation using a Likens-Nickerson apparatus with continuous extraction with dichloromethane	α-pinene, α-phellandrene, germacrene D	*A. niger*	625		
*Rudbeckia laciniata* L.	Hydro-distillation using a Likens-Nickerson apparatus with continuous extraction with dichloromethane	α-pinene, β-pinene, myrcene, limonene	*A. niger*	625		
*Solidago altissima* L.	Hydro-distillation using a Likens-Nickerson apparatus with continuous extraction with dichloromethane	α-pinene, sabinene, myrcene, bornyl acetate, germacrene D	*A. niger*	625		
*Xanthium* *strumarium* L.	Hydro-distillation using a Likens-Nickerson apparatus with continuous extraction with dichloromethane	(2E)-hexenal, myrcene, limonene, germacrene D	*A. niger*	625		
				**MIC (mg/mL)**		
*Myristica fragrans*	Hydro-distillation	Elemicin, myristicine, thujanol, methyl eugenol, safrole	*A. flavus*	2.75		[118]
				**MIC (mg/mL)**	**ZOI (mm)**	
*Pulicaria crispa* (Forsk.) Oliv.	Hydro-distillation	Carvone, caryophyllene, neryl (S)-2-methylbutanoate, 1,4-ditert-butylbenzene	*A. niger*	6.25	21	[119]
*Pulicaria undulata* (L.) C.A.Mey	Hydro-distillation	Bicyclo, camphor, thymyl acetate, azulenol	*A. niger*	6.25	22	
				**MIC (μg/mL)**	**ZOI (mm)**	
*Thymus decussatus*	Hydro-distillation Microwave-assisted extraction	Carvacrol, p-cymene	*A. niger*	**0.49**	47.00 48.00	[100]
*Teucrium polium*	Hydro-distillation Microwave-assisted extraction	Aromadendrene, germacrene-D, β-muurolene, α-muurolene, δ-cadinene, germacrene d-4-ol, τ-muurolol, α-cadinol, alloaromadendrene oxide, 6-epi-shyobunol)	*A. niger*	n.a.	n.a.	
				**MGI**		
*Thymus kotschyanus*	Clevenger hydro-distillation	p-cymene, γ-terpinene, thymol, carvacrol	*A. niger*	250 ppm (partial inhibition) ≥500 ppm (fungicidal)		[120]
				**MIC (μg/mL)**		
*Centaurea scoparia*	Hydro-distillation Microwave-assisted extraction	Trans-caryophyllene, spathulenol, theaspirane A, theaspirane B, methyl hexadecanoate	*A. niger*	n.a.		[121]
*Centaurea calcitrapa,*	Hydro-distillation Microwave-assisted extraction	Boronal, spathulenol, α-cadinol, aromadendrene oxide-2, α-costol, phytol, paeonol, arachidic acid	*A. niger*	n.a.		
*Centaurea glomerata*	Hydro-distillation Microwave-assisted extraction	Spathulenol, α-guaiol, eudesmol, aromadendrene oxide-2, α-costol, geranylterpinene, phytol tricosane, 2-phenylethyl octadecanoate	*A. niger*	n.a.		
*Centaurea lipii*	Hydro-distillation Microwave-assisted extraction	Isothujone, spathulenol, torreyol, aromadendrene oxide-2, icosane, henicosane, 2-phenylethyl octadecanoate	*A. niger*	1000		
*Centaurea alexandrina*	Hydro-distillation Microwave-assisted extraction	1,8-cineole, isothujone, spathulenol, torreyol, aromadendrene oxide-1, 13-epi-manool, thunbergol, phytol, henicosane, methyl arachidonate, arachidic acid, (Z)-9-octadecenamide, 2-phenylethyl octadecanoate	*A. niger*	n.a.		
				**MGI (%)**		
*Coleus aromaticus*	Microwave-assisted solvent-free extraction	Thymol, thymoquinone, creosol, linalool, p-cymene-2,5-diol, p-cymene	*A. niger*	79.63 (poisoned food test)70.45 (disc diffusion assay)		[122]
				**MIC (μg/mL)**	**ZOI (mm)**	
*Dracocephalum* *kotschyi* Boiss. (Lamiaceae)	Clevenger hydro-distillation	α-pinene, limonene, α-campholenal, cyclohexylallene, Z-citral=neral, geraniol, (E)-citral, methyl geranate	*A. niger*	2000 (cultivated plants) 500 (wild plants)	15 (cultivated plants)	[123]
			*A. brasiliensis*	2000 (cultivated plants) 250 (wild plants)	25 (cultivated plants) 26 (wild plants)	
				**MIC (μg/mL)**	**ZOI (mm)**	
*Asteriscus graveolens* (Forssk.) Less.	Clevenger hydro-distillation	p-cineole, α-thujone, camphor, carvacrol	*A. niger* *A. flavus*	**24.50** **23.74**	17.01 16.76	[102]

The bold value for antifungal activity indicates noteworthy activity (MIC ≤ 0.1 mg/mL). “-”: not determined; AI: activity index; GI: growth inhibition; MFC: minimum fungicidal concentration; MGI: mycelia growth inhibition; MIC: minimum inhibitory concentration; n.a.: not active; ZOI: zone of inhibition.

Niosome-encapsulated EOs of *Satureja montana* and *Origanum virens* were formulated and tested against *A. flavus*. Initially, after the incubation for 7 days, the colony forming units per gram (CFU/g) values of pure EOs were lower than the niosome-encapsulated EOs. However, the pure EOs lost their antifungal effect over time and the fungal growth increased with higher CFU/g values after incubated for 21 days, in contrast to the decreased fungal growth and lower CFU/g values for niosome-encapsulated EOs [124]. Poly(ε-caprolactone) (PCL) nanocapsules containing EOs of *Origanum vulgare* and *Thymus capitatus* were reported to be active against various fungal strains including *A. fumigatus* and *A. flavus*, with MIC and MFC values two to four times lower compared to the pure EOs, indicating the improved antifungal activity of the EOs by nanoencapsulation. Encapsulation of *Cuminum cyminum* EOs by chitosan-caffeic acid nanogel considerably improved its performance with a lower MIC against *A. flavus* compared with free EO under sealed condition (350 ppm vs. 650 ppm) and non-sealed condition (800 ppm vs. >1000 ppm) [125]. Encapsulation enables a lower dose of EOs to be used to achieve the same antifungal effect, thus reducing the toxicity effects associated with high dose [126]. The antifungal effects of other plant crude extracts against *Aspergillus* spp. are presented in Supplementary Table S1.

## 5. Novel Delivery Systems for Plants Extracts against *Aspergillus* spp.

The bioavailability of plant bioactives is limited by poor solubility, poor stability due to gastric and colonic acidity, poor metabolism by gut microflora, poor absorption across the intestinal wall and active efflux mechanism as well as first-pass metabolic effects, failing clinical studies [127]. Novel drug delivery systems and carriers for herbal medicines have been developed with the aim of delivering the phytochemicals to the site of action at a rate according to the body’s needs throughout the treatment.

Nanocarrier delivery has gained great attention to overcome the physicochemical and pharmacokinetic limitations of phytochemicals, enhance controlled release as well as improve the efficacy of bioactivities [128]. *Illicium verum* EO with anethole as the major compound has reported MIC value of 0.7 μL/mL against aflatoxigenic strain *A. flavus* LHP-PV-1 as well as promising free radical scavenging activity and favourable safety profile with high LD_50_ value (11257.14 μL/kg) [115]. Enhanced antifungal potency was demonstrated when the EO was nanoencapsulated. The MIC values of both lyophilized and non-lyophilized *I. verum* EO-loaded chitosan nanoparticles (0.3 and 0.4 μL/mL, respectively) were lower than the EO in addition to the lower value of minimum aflatoxin B1 inhibitory concentration (MAIC) against *A. flavus* LHP-PV-1. Strong antifungal, antiaflatoxigenic and antioxidant activities of the encapsulated EO suggest its use as a plant-based preservative and shelf-life enhancer of food items, which must be confirmed in future long-term studies.

The use of plant extracts in the green synthesis of metallic nanoparticles may produce new nanomaterial with more potent and/or novel biological activities due to the synergistic effect [90]. Using resazurin microtiter assay, the estimated MIC values of *M. oleifera* extract against *C. albicans* and *C. glabrata* were reduced from 125 and 250 μg/mL, respectively, to 62.5 μg/mL by the synthesized *M. oleifera* bismuth nanoparticles (NPs), indicating a synergistic effect [90]. However, the MIC values of the NPs against *A. niger* and *A. flavus* (250 μg/mL) were higher compared with the plant extract (62.5 μg/mL). Another study also demonstrated the poorer antifungal activity of *M. oleifera* copper NPs against *A. niger* and *A. flavus* (125 μg/mL) compared with the *M. oleifera* leaves extract (62.5 μg/mL) [91]. Thus, the synthesized metallic nanoparticles may be more useful in the treatment of Candida infections but not those caused by *Aspergillus* spp.

## 6. Conclusions and Future Prospective

*Aspergillus* spp. poses a life threat to society, and particularly immunocompromised patients, since many strains have shown resistance towards the existing antifungal agents. Medicinal plants provide new hope in the fight against *Aspergillus* infections. An increased action to identify and isolate new bioactive compounds that may potentially be used as a new treatment against *Aspergillus* infections is imperative. In this context, a large number of plant extracts and their constituents have shown potential as a therapeutic alternative. However, the implementation of plant extracts as a part of the therapeutic regimen is still challenging. Although in vitro studies showed promising results, in vivo studies should be conducted in order to establish a valid correlation with the in vitro data. Standardisation of the active compound in the plant materials is also necessary to ensure a reproducible and consistent antifungal activity in each batch of production. Due to problems associated with the bioavailability of plant extracts, studies on the novel delivery system of the plant extracts are highly warranted to enhance the therapeutic potential of these extracts. Additionally, interactions among different plant extracts, compounds, or antifungal agents should also be studied to maximise the antifungal efficacy while minimizing toxicity. This review summarizes the information on plant extracts with potent anti-*Aspergillus* effects that can aid in a faster and more efficient process in the development of new plant-derived antifungal agents against *Aspergillus* infections.

## Supplementary Materials

The following supporting information can be downloaded at: https://www.mdpi.com/article/10.3390/plants11223009/s1, Table S1. Antifungal effects of other plant crude extracts against *Aspergillus* spp. References [60,129,130,131,132,133,134,135,136,137,138,139] are cited in the supplementary materials.
