# Supplementary material for: Plant as an Alternative Source of Antifungals against Aspergillus Infections: A Review
Source: Plants (Basel). 2022 Nov 8;11(22):3009. doi: 10.3390/plants11223009 (PMC9697101; doi:10.3390/plants11223009)
Supplement: Supplementary file 1 [file plants-11-03009-s001.zip › plants-1949936-supplementary.pdf]

Supplementary Material

Table S1. Antifungal effects of other plant crude extracts against *Aspergillus* spp.

Plant	Family	Extraction Solvent	Phytochemical Constituents/ Main Compounds Identified	*Aspergillus* Species	Antifungal Activity			Reference
					**ZOI at 150 μL (mm)**			
Eucomis bicolor	Asparagaceae	Acetone (70%)	-	*A. clavatus*	18			[1]
				A. niger	n.a.			
					**MIC (g/L)**	**MFC (g/L)**	**ZOI at 400 g/L (mm)**	
Phlomis olivieri	Lamiaceae	Methanol (extract)	-	*A. flavus*	n.a.	n.a.	n.a.	[2]
				A. fumigatus	n.a.	n.a.	n.a.	
		Petroleum ether (fraction)	-	*A. flavus*	1	4	19.4	
				A. fumigatus	0.5	2	18.3	
		Chloroform (fraction)	-	*A. flavus*	1	2	21.4	
				A. fumigatus	2	4	21.2	
		Ethyl acetate (fraction)	-	*A. flavus*	0.5	2	22.4	
				A. fumigatus	1	4	22.2	
		Water	-	*A. flavus*	n.a.	n.a.	n.a.	
				A. fumigatus	n.a.	n.a.	n.a.	
					**ZOI (mm)**			
Usnea florida (L.)	Parmeliaceae	Methanol	Thamnolic acid	*A. niger*	n.a.			[3]
		Chloroform		*A. flavus*				
		Acetone		*A. parasiticus*				
				A. fumigatus				
					**ZOI at 1000 μg/disc (mm)**			
Atrichum undulatum (Hedw.) P. Beauv	Polytrichaceae	Water	-	*A. fumigatus*	10.9			[4]
		Ethanol (99%)			14.8			
					**GI at 1 mg/mL (%)**			
Bergenia ciliata Sternb	Saxifragaceae	Methanolic	Steroids, terpenoids, tannins, flavonoids	*A. flavus*	n.a.			[5]
		n-Hexane		*A. niger*				

		Chloroform						
		Ethyl acetate						
						**GI (%)**		
Portulaca oleracea L.	Portulacaceae	Ethanol (90%)	-	*A. flavus*		73.47-76.47		[6]
					**MIC (mg/mL)**	**MGI at MIC (%)**	**Total Activity (mL/g)**	
Acokanthera oppositifolia	Apocynaceae	Acetone (30%)	-	*A. flavus*	**0.16**	**12.29**	**208**	[7]
				A. ochraceous	**1.25**	**1.32**	**27**	
Apodytes dimidiata	Metteniusaceae	Acetone (30%)	-	*A. flavus*	0.16	**30.79**	**1104**	
				A. ochraceous	1.25	4.61	141	
Artemesia afra	Asteraceae	Acetone (30%)	-	*A. flavus*	0.16	13.62	**1000**	
				A. ochraceous	0.31	18.57	516	
Bauhinia galpini	Fabaceae	Acetone (30%)	-	*A. flavus*	0.16	3	396	
				A. ochraceous	0.1	3.38	633	
Brachylaena discolor	Asteraceae	Acetone (30%)	-	*A. flavus*	0.16	8.64	271	
				A. ochraceous	2.5	13.47	17	
Breonadia salicina	Rubiaceae	Acetone (30%)	-	*A. flavus*	0.16	5.75	854	
				A. ochraceous	1.25	**26.33**	109	
Capparis tamentosa	Capparaceae	Acetone (30%)	-	*A. flavus*	0.31	7.97	280	
				A. ochraceous	0.31	9.04	280	
Combretum caffrum	Combretaceae	Acetone (30%)	-	*A. flavus*	0.16	13.64	521	
				A. ochraceous	0.16	5	521	
Dracaena mannii	Asparagaceae	Acetone (30%)	-	*A. flavus*	0.31	21.04	226	
				A. ochraceous	0.63	**27.99**	111	
Ficus natelensis	Moraceae	Acetone (30%)	-	*A. flavus*	2.5	1.86	13	
				A. ochraceous	0.31	11.54	108	
Harpephyllum caffrum	Anacardiaceae	Acetone (30%)	-	*A. flavus*	0.63	4.73	196	
				A. ochraceous	0.78	16.29	158	
Heteromorpha arborescens	Apiaceae	Acetone (30%)	-	*A. flavus*	0.63	**32.13**	222	
				A. ochraceous	0.31	16.11	452	
Kirkia wilmsii	Kirkiaceae	Acetone (30%)	-	*A. flavus*	0.16	12.09	**1042**	
				A. ochraceous	2.5	7.34	67	
	Celastraceae		-	*A. flavus*	0.16	8.35	500	

Maytenus undata		Acetone (30%)		*A. ochraceous*	**0.08**	10.35	1000	
Millettia grandis	Fabaceae	Acetone (30%)	-	*A. flavus*	0.16	8.26	500	
				A. ochraceous	0.2	14.58	400	
Mystroxylon aethiopicum	Celastraceae	Acetone (30%)	-	*A. flavus*	0.16	**38.15**	542	
				A. ochraceous	0.31	11.28	280	
Ricinus communis	Euphorbiaceae	Acetone (30%)	-	*A. flavus*	**0.08**	9.38	500	
				A. ochraceous	0.2	3.65	400	
Solanum aculeastrum	Solanaceae	Acetone (30%)	-	*A. flavus*	0.16	11.4	438	
				A. ochraceous	0.39	15.36	179	
Spirostachys africana	Euphorbiaceae	Acetone (30%)	-	*A. flavus*	0.16	19.17	**1167**	
				A. ochraceous	0.16	**31.04**	**1167**	
Strychnos mitis	Loganiaceae	Acetone (30%)	-	*A. flavus*	1.25	11.35	131	
				A. ochraceous	0.31	24.69	527	
Warburgia salutaris	Canellaceae	Acetone (30%)	-	*A. flavus*	0.78	**29.73**	154	
				A. ochraceous	0.13	**28.59**	923	
Xylotheca kraussiana	Achariaceae	Acetone (30%)	-	*A. flavus*	0.78	5.52	449	
				A. ochraceous	2.5	7.34	140	
Zanthoxylum capense	Rutaceae	Acetone (30%)	-	*A. flavus*	0.78	12.54	103	
				A. ochraceous	0.16	8	500	
Ziziphus nummularia	Rhamnaceae	Methanol (90%) followed by sequential fractionation using chloroform, n-hexane, ethyl acetate and water	Flavonoids, steroids/triterpenoid, alkaloids, tannins, saponins	*A. niger*	n.a. (MIC >10 mg/mL)			[8]
					**MIC (μg/mL)**			
Ammi majus L.	Apiaceae	Ethanol (70%)	-	*A. fumigatus*	>100			[9]
					**MIC (mg/mL)**		**ZOI (mm)**	
Curcuma zedoaria	Zingiberaceae	n-hexane	-	*A. niger*	n.a.		n.a.	[10]
				A. flavus	n.a.		n.a.	

		Chloroform	-	*A. niger*	n.a.	n.a.	
				A. flavus	274	12	
		Ethyl acetate	-	*A. niger*	275	12	
				A. flavus	274	12	
		n-butanol	-	*A. niger*	252	14	
				A. flavus	278	12	
		Water	-	*A. niger*	n.a.	n.a.	
				A. flavus	275	12	
Dicliptera bupleuroides	Acanthaceae	n-hexane	-	*A. niger*	n.a.	n.a.	
				A. flavus	n.a.	n.a.	
		Chloroform	-	*A. niger*	251	14	
				A. flavus	278	12	
		Ethyl acetate	-	*A. niger*	276	12	
				A. flavus	274	12	
		n-butanol	-	*A. niger*	252	14	
				A. flavus	276	12	
		Water	-	*A. niger*	275	12	
				A. flavus	276	12	
Fumaria indica	Papaveraceae	n-hexane	-	*A. niger*	n.a.	n.a.	
				A. flavus	387	4	
		Chloroform	-	*A. niger*	251	14	
				A. flavus	155	20	
		Ethyl acetate	-	*A. niger*	275	12	
				A. flavus	251	14	
		n-butanol	-	*A. niger*	n.a.	n.a.	
				A. flavus	276	12	
		Water	-	*A. niger*	275	12	
				A. flavus	251	14	
					**MIC (mg/mL)**		
Alternanthera sessilis	Amaranthaceae	Sequential extraction using hexane, chloroform, ethyl acetate,	Alkaloids, flavonoids, cardiac glycosides, saponins, sterols, terpenoids	*A. fumigatus*	n.a. (>2.50)		[11]

		ethanol, methanol, and distilled water			
Catunaregam spinosa	Rubiaceae	Sequential extraction using hexane, chloroform, ethyl acetate, ethanol, methanol, and distilled water	Iridoid (10-methylixoside)	*A. fumigatus*	n.a. (>2.50)
Ipomoea aquatica	Convolvulaceae	Sequential extraction using hexane, chloroform, ethyl acetate, ethanol, methanol, and distilled water	Favonoids (quercetin, quercetin 3′-methylether, quercetin 4′-methylether, 3α-7β-O-D-diglycopyranosyl-dihydroquercetin), anthocyanins, carotenoids	*A. fumigatus*	n.a. (>2.50)
Tradescantia spathacea	Commelinaceae	Sequential extraction using hexane, chloroform, ethyl acetate, ethanol, methanol, and distilled water	Alkaloids, cardiac glycosides, flavonoids, saponins, steroids, tannins, terpenoids	*A. fumigatus*	n.a. (>2.50)
					**MIC (mg/mL)**

Tulbaghia violacea (Harv)	Amaryllidaceae	Deionised water	-	*A. flavus*	15	[12]

The bold value for antifungal activity indicates noteworthy activity (MIC ≤ 0.1 mg/mL).
"-": not determined; AI: activity index; GI: growth inhibition; MFC: minimum fungicidal concentration; MGI: mycelia growth inhibition; MIC: minimum inhibitory concentration; n.a.: not active; ZOI: zone of inhibition

References

1. Mizielińska, M.; Salachna, P.; Ordon, M.; Łopusiewicz, Ł. Antimicrobial activity of water and acetone extracts of some *Eucomis* taxa. *Asian Pac. J. Trop. Med.* **2017**, *10*, 892–895, doi:10.1016/j.apjtm.2017.08.018.
2. Asgarpanah, J.; Hashemi, S.J.; Hashemi, E.; Askari, K. *In vitro* antifungal activity of some traditional Persian medicinal plants on pathogenic fungi. *Chin. J. Integr. Med.* **2017**, *23*, 433–437, doi:10.1007/s11655-015-2181-7.
3. Cankılıç, M.Y.; Sarıözlü, N.Y.; Candan, M.; Tay, F. Screening of antibacterial, antituberculosis and antifungal effects of lichen *Usnea florida* and its thamnolic acid constituent. *Biomed. Res.* **2017**, *28*, 3108–3113.
4. Saxena, K.; Yadav, U. *In vitro* assessment of antimicrobial activity of aqueous and alcoholic extracts of moss *Atrichum undulatum* (Hedw.) P. Beauv. *Physiol. Mol. Biol. Plants* **2018**, *24*, 1203–1208, doi:10.1007/s12298-018-0589-1.
5. Khan, A.; Jan, G.; Khan, A.; Jan, F.G.; Danish, M. Evaluation of antioxidant and antimicrobial activities of *Bergenia ciliata* Sternb (Rhizome) crude extract and fractions. *Pak. J. Pharm. Sci.* **2018**, *31*, 31–35.
6. Li, X.M.; Liu, J.; Pan, F.F.; Yang, P.L. Effect of quality control on the antiproliferative activity of the extract from *Portulaca oleracea* L. in *Aspergillus flavus*. *Biomed. Chromatogr.* **2018**, *32*, doi:10.1002/bmc.4354.
7. Dikhoba, P.M.; Mongalo, N.I.; Elgorashi, E.E.; Makhafola, T.J. Antifungal and anti-mycotoxigenic activity of selected South African medicinal plants species. *Heliyon* **2019**, *5*, e02668, doi:10.1016/j.heliyon.2019.e02668.
8. Ullah, A.; Mustafa, G.; Hanif, M.; Mohibullah, M.; Bakhsh, S.; Rashid, S.A.; Zaman, A.; Rehman, F.; Khan, B.A.; Amin, A. Antibacterial and antibiofilm properties of traditional medicinal plant from Sheikh Buddin range. *Pak. J. Pharm. Sci.* **2019**, *32*, 1313–1319.
9. Fathallah, N.; Raafat, M.M.; Issa, M.Y.; Abdel-Aziz, M.M.; Bishr, M.; Abdelkawy, M.A.; Salama, O. Bio-guided fractionation of prenylated benzaldehyde derivatives as potent antimicrobial and antibiofilm from ammi majus l. fruits-associated aspergillus amstelodami. *Molecules* **2019**, *24*, doi:10.3390/molecules24224118.
10. Riaz, T.; Abbasi, M.A.; Aziz-ur-Rehman; Shazadi, T.; Shahid, M. Assessment of *Fumaria indica*, *Dicliptera bupleuroides* and *Curcuma zedoaria* for their antimicrobial and hemolytic effects. *Pak. J. Pharm. Sci.* **2019**, *32*, 697–702.
11. Oon, Y.N.; Chen, R.J.; Kuan, J.M.; Sit, N.W. Bioactivity of medicinal plant extracts against human fungal pathogens and evaluation of toxicity using vero cells. *Trop. Biomed.* **2021**, *38*, 469–475, doi:10.47665/tb.38.3.090.
12. omai, B.M.; Belewa, V.; Frost, C. *Tulbaghia violacea* (Harv) exerts its antifungal activity by reducing ergosterol production in *Aspergillus flavus*. *Curr. Microbiol.* **2021**, *78*, 2989–2997, doi:10.1007/s00284-021-02546-1.
